# Prevalence of ORAL Surveillance-like Cardiovascular Risk Factors in Adults Patients With Inflammatory Bowel Diseases: National Estimates From the United States

**DOI:** 10.1016/j.gastha.2025.100723

**Published:** 2025-06-14

**Authors:** Erin Song, Yuchen Qi, Siddharth Singh

**Affiliations:** 1 Division of Gastroenterology, Department of Medicine, University of California San Diego, La Jolla, California; 2 Biostatistics and Bioinformatics, Herbert Wertheim School of Public Health, University of California San Diego, La Jolla, California; 3 Division of Biomedical Informatics, Department of Medicine, University of California San Diego, La Jolla, California

Janus kinase inhibitors (JAKi), particularly upadacitinib, are among the most efficacious therapies for patients with inflammatory bowel diseases (IBDs), particularly ulcerative colitis.^1^ However, in 2019, regulatory agencies modified the label for JAKi based on the ORAL Surveillance Trial.^2^ This noninferiority trial compared the safety of tofacitinib (5 mg or 10 mg twice daily dosing) versus tumor necrosis factor (TNF)-α antagonist in patients with rheumatoid arthritis who were aged ≥ 50 and had at least 1 additional cardiovascular risk factor including current smoker, hypertension, diabetes, coronary artery disease (CAD), high-density lipoprotein (HDL) cholesterol level <40 mg/dL, family history of premature coronary heart disease and/or extra-articular rheumatoid arthritis. Over a median follow-up of 4 years, tofacitinib was associated with a higher risk of major adverse cardiac events (3.4% vs 2.5%; hazard ratio, 1.33), venous thromboembolism, cancer and death. Based on this study, the US Food and Drug Administration changed JAKi labeling across all indications, restricting its use in patients with prior failure or intolerance to TNF antagonists.^3^ The European Medicines Agency (EMA) also recommended cautious JAKi use as first-line in patients at cardiovascular risk, including age ≥ 65 years, smokers, history of cardiovascular disease, or cancer.^4^

There has been limited population-based evaluation of how many patients with IBD would be at high risk of cardiovascular events, akin to the population included in ORAL Surveillance, and hence impacted by these restrictions. We utilized the National Health Interview Survey (NHIS) 2023 to estimate prevalence of ORAL Surveillance-like cardiovascular risk factors in patients with IBD.^5^

NHIS is an annual household survey compiled by the National Center for Health Statistics/Center for Disease Control & Prevention using multistage probability sampling to monitor the health of noninstitutionalized, US population, on a broad range of health topics, by collecting data on health status, health-care access, and health behaviors. This includes information on chronic conditions, mental health, health insurance coverage, use of health-care services, and behaviors like smoking or physical activity. NHIS data are publicly available as deidentified data. While the survey is not specifically designed to study patients with IBD, in NHIS 2023, patients were identified as having a diagnosis of IBD based on an affirmative response to the question, “Have you ever been told by a doctor or other health professional that you had Crohn’s disease or ulcerative colitis?” We conducted weighted descriptive analyses to estimate the number of ORAL Surveillance-like patients with IBD based on different combinations of risk factors in the entire US adult population.^2^ We estimated the number of patients with IBD with age ≥50 years that met at least one cardiovascular risk factors included in the ORAL Surveillance Trial. Risk factors were based on ever being diagnosed with hypertension, diabetes, current smoking and personal history of CAD. We used self-reported diagnosis of hyperlipidemia as a surrogate for HDL <40 mg/dL. The survey did not include questions on family history of premature CAD. Less than 5% data were missing and no imputation was performed. We conducted sensitivity analysis using alternative definitions of risk factors (hypertension in the last 12 months, rather than ever; self-reported diabetes in the last 12 months and on oral hypoglycemic agents or insulin; diagnosed with hyperlipidemia in the last 12 months). We also captured proportion of patients taking aspirin. All statistical analyses were implemented in R version 4.2.2. (R Project for Statistical Computing).

The data underlying this article are available in the NHIS website (at https://www.cdc.gov/nchs/nhis/documentation/2023-nhis.html).

**Figure 1:**
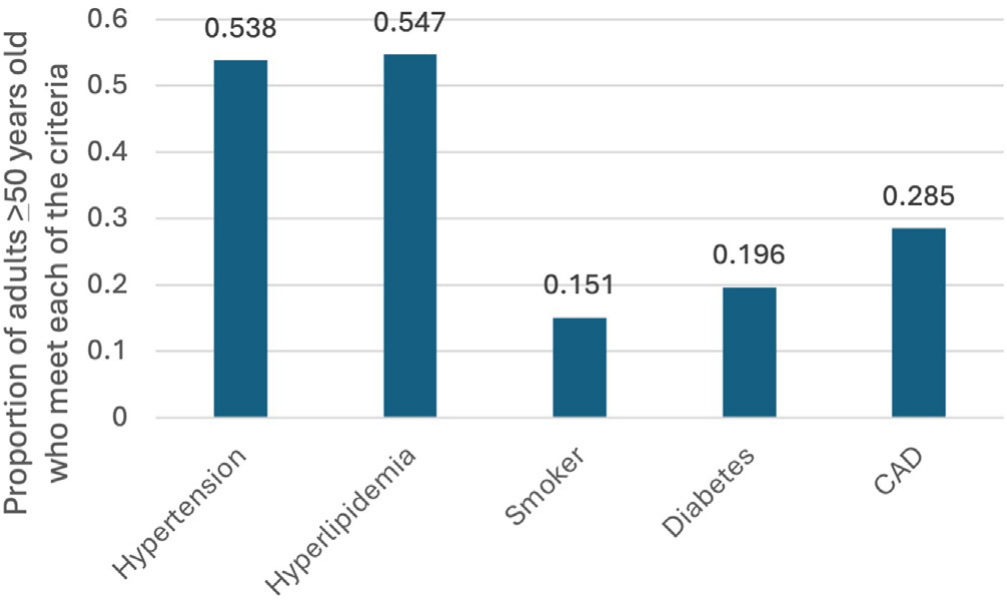
Prevalence of different cardiovascular risk factors in adults with IBD ≥50 years using NHIS 2023.

Of the total 572 patients with IBD identified in the survey (estimated US population living with IBD of 4.1 million), 49.8% were ≥50 years and met at least 1 cardiovascular risk factor included in the ORAL Surveillance Trial. Among these patients, 53.8% reported hypertension, 54.7% reported high cholesterol, 15.1% were current smokers, 19.6% reported diabetes, and 28.5% had CAD (Figure 1). When using a stricter definition of risk factors, 44.5% patients with IBD were ≥50 years and met at least 1 cardiovascular risk factor included in the ORAL Surveillance Trial. On analyses of adults with IBD ≥50 years, 20.5% reported no cardiovascular risk factors, whereas 27.7% reported 1, 24.1% reported 2, and 28.7% reported 3 or more. Aspirin use was reported by 41.7% (whether self-initiated or prescribed); among those with at least one cardiovascular risk factor, 44.7% were taking aspirin. Using the EMA criteria, 21% patients with IBD were ≥50 years, 15% were current or previous smokers, and 1.7% met both criteria.

Based on US national estimates, approximately half the patients living with IBD would meet criteria for ORAL Surveillance-like cardiovascular risk factors, with nearly 80% of those aged ≥50 years having at least one risk factor and 29% with three or more risk factors. In contrast, when using EMA criteria, approximately one-third patients with IBD were ≥65 years and/or ever smokers. These findings suggest that a substantial proportion of adults living with IBD may be deemed at high cardiovascular risk where JAKi use would be restricted when following regulatory guidance. These findings also highlight the pressing need to estimate whether the increased cardiovascular risk observed with tofacitinib vs TNF antagonists in patients with rheumatoid arthritis also applies to other JAKi, especially selective JAK1 inhibitors and to patients with IBD.

Our findings align with other surveys on prevalence of cardiovascular risk in patients with IBD. For example, a multicenter study of 1071 patients with ulcerative colitis (median age 44) from 35 referral centers in Belgium and France found that 58% of those over age 45 had at least 1 cardiovascular risk factor.^6^ In comparison, the mean age of adults with IBD in NHIS 2023 was 56. Their findings were based on self-selected group of responders to an optional survey, compared with the NHIS where complex multistage probability sampling allows extrapolation to the entire noninstitutionalized US population. Limitations include self-reported diagnosis of IBD and of putative cardiovascular risk factors without validation. NHIS did not capture family history of premature CAD or HDL cholesterol levels. The NHIS may over-represent older adults, which limits generalizability of our findings to younger patients with IBD.

In summary, using a US national survey, approximately half the patients living with IBD would meet the criteria for ORAL Surveillance-like cardiovascular risk factors where JAKi use would be restricted. A more comprehensive understanding of comparative cardiovascular risk with JAKi and other agents, in patients with IBD is urgently needed to inform treatment approach.
